# A Rasch analysis of emerging adults’ health motivation questionnaire in higher education context

**DOI:** 10.1371/journal.pone.0248389

**Published:** 2021-03-15

**Authors:** Min An, Xiaofei Yu

**Affiliations:** 1 School of Education, Linyi University, Linyi, China; 2 School of Education, Wenzhou University, Wenzhou, China; 3 Linyi University Library, Linyi, China; University of Copenhagen, DENMARK

## Abstract

**Objective:**

The College Students’ Health Motivation Questionnaire (CSHM-Q) is used to measure motivation for a healthy lifestyle among emerging adults. This study sought to validate the CSHM-Q using the Rasch measurement model.

**Methods:**

322 participants were recruited based on a convenience sampling method. The Rasch analysis was carried out using the RUMM2030 software.

**Results:**

Local item dependency was accommodated using the ‘super item’ approach. Disordered thresholds were resolved by collapsing some response options. After modification, each component of the CSHM-Q showed acceptable overall fit, item and person fit, internal consistency, and targeting. Unidimensionality was supported at the subscale level. Items did not exhibit disordered threshold, local item dependency, or differential item functioning. Transforming tables were also created to help convert the raw score into an interval scale.

**Conclusions:**

Results of the Rasch analysis supported the interval scale measurement properties of the CSHM-Q and offered health education researchers an instrument to measure emerging adults’ health motivation in the higher education context.

## Introduction

Motivation, particularly intrinsic motivation, plays a key role in one’s adoption of a healthy lifestyle [1, 2]. In the area of health education and promotion, motivation has been advocated as a crucial variable in a number of theoretical models (e.g., the Theory of Reasoned Action, the Theory of Planned Behavior, and Pender’s Health Promotion Model [3]) aiming to promote health-related behaviors.

A number of scales have been developed to measure motivations for health-promoting behaviors [4]. Among these scales, the College Students’ Health Motivation Questionnaire (CSHM-Q) is a novel and generic instrument to assess motivation for a healthy lifestyle among emerging adults. Acceptable psychometric properties of the CSHM-Q have been reported by a previous study based on methods of classical test theory [5]. Parallel analysis and exploratory factor analysis showed that it has a 3-component structure with 16 items in total. Please refer to the method section for a brief description of the CSHM-Q.

Since the CSHM-Q was developed and validated based on approaches of classical test theory only, and a known weakness of traditional test theory is that the estimation of psychometric properties is sample dependent [6], the current study aimed to further validate the original CSHM-Q through the application of a modern psychometric approach—Rasch analysis.

### The Rasch model and Rasch analysis

The Rasch model was first developed by Georg Rasch [7]. It is an unidimensional measurement model, with a set of requirements to satisfy fundamental measurement. Unlike other statistical models which give emphasis to explaining variance, the Rasch model forms a template for fundamental measurement. Although the Rasch model is mathematically identical to a one parameter model in IRT, it is regarded as incompatible to other IRT models with its emphasis upon model supremacy [8]. In Rasch analysis, if the observed data do not fit the model, the aim would be to adapt the data to fit the Rasch model; in IRT analysis, conversely, the aim is to find a more suitable model to fit the data.

The Rasch model assumes that the probability of a test-taker affirming a given item is a logistic function of the difference between the item difficulty and person ability on a same logit metric.

The Rasch model for dichotomous data can be described as:

$$P_{ni}=\frac{{\text{e}}^{\left(\beta_{n}-\delta_{i}\right)}}{1+{\text{e}}^{\left(\beta_{n}-\delta_{i}\right)}}$$ (1)

Where *P*_*ni*_ is the probability that person *n* will endorse the item *i*; *β*_*n*_ is person n’s estimated ability, or the level of health motivation in the present research setting; *δ*_*i*_ is the estimated difficulty of item i, or the level of health motivation expressed by item i in the present context. Using this formula, person’s ability and item’s difficulty are logarithmically transformed and plotted on a same continuum measured by *logit* as a common unit. Therefore, *β*_*n*_ − *δ*_*i*_ is the logit distance between person ability and item difficulty on that continuum, and the dichotomous form of Rasch model can also be expressed as:

$$\text{ln}(\frac{P_{\text{ni}}}{1-P_{\text{ni}}})=\beta_{n}-\delta_{i}$$ (2)

The Rasch model for polytomous data can be expressed as:

$$\text{ln}(\frac{P_{nij}}{1-P_{nij-1}})=\beta_{n}-\delta_{i}-\tau_{j}$$ (3)

Compared to the dichotomous form (2), the additional τ_*j*_ denotes the threshold between two adjacent categories. This model is known as the Rasch rating scale model (RSM). In RSM, items have the same rating scale structure—every item shares the same number of response categories and the distances between threshold parameters are maintained across all items. In addition to RSM, the partial credit model (PCM) does not hold constraints on threshold parameters and allowing them to vary—items can have different number of response categories and unequal-distance between each threshold parameter [9]. The PCM can be expressed as:

$$\text{ln}(\frac{P_{nij}}{1-P_{nij-1}})=\beta_{n}-\delta_{ij}$$ (4)

Unlike CTT and IRT, Rasch analysis can produce item-distribution free and person-distribution free measurement [6, 9]. That is, the measurement of any person’s trait is independent of the dispersion of the set of items used to measure that trait; item calibration is independent of the distribution of the ability in the sample of persons who take the test. This unique advantage is often stated as specific objectivity, which allows separate person and item estimates measured on the same logit metric. This means that difficult items will always have less endorse rates irrespective of test-takers who are administered. On the other hand, the person calibration is test independent, meaning that proficient test-takers will always have a better performance than those who are less proficient irrespective of what tests they are facing. When data are fitted to the Rasch model and meet its expectations, a total summed score becomes more valid because linear measures can be constructed from counts of qualitatively ordered observations [10].

## Participants and methods

### Study population

Data were obtained from participants at Linyi University, China, based on a convenience sampling strategy. Data cleaning was performed after data collection to improve data quality before subsequent statistical analyses. Data redundancy, inconsistent responses, extreme categories, and uniform response vectors were examined. Little’s test [11] showed the data appeared to be missing completely at random (MCAR), which means the probability of missing data on a variable is unrelated to any other measured variable, therefore data with missing values were removed. After data cleaning, 322 cases were retained for following Rasch analyses.

According to Linacre [12], the minimum sample size for Rasch analysis is affected by scale targeting. Wright and Stone [13] performed a Rasch analysis based on a sample of 35 participants and 18 items. As Linacre [12] indicated, 243 cases will be enough to precisely estimate items and persons’ locations regardless of scale targeting. Therefore, a sample size of 322 was sufficient in the present study. This sample included 87 males and 235 females (mean age = 20.02 years, standard deviation = 1.43). Most of them were first- or second-year college students. Their family residence included urban, suburban, and countryside. Demographic information of study participants is presented in Table 1.

**Table 1:** Demographic information of the study sample (n = 322).

Demographics	N (%)
**Sex**	
**Female**	87 (27.0%)
** Male**	235 (73.0%)
**Age**	
** 18**	42 (13.0%)
** 19**	87 (27.0%)
** 20**	89 (27.6%)
** 21**	50 (15.5%)
** 22**	36 (11.2%)
** 23**	18 (5.6%)
**College year**	
** First year**	140 (43.5%)
** Second year**	130 (40.4%)
** Third year**	25 (7.8%)
** Fourth year**	23 (7.1%)
**Family residence**	
** Urban**	128 (39.8%)
** Suburban**	66 (20.5%)
** Countryside**	128 (39.8%)

### Ethical approval

Ethical approval was obtained from the Linyi University Ethical Review Committee. Before data collection took place, the nature, purpose, and ethical issues of the study were explained to the participants, all participants signed a consent form and finished the questionnaire anonymously.

### Instrument

The CSHM-Q [5] was developed based on the Self-Determination Theory [14]. It is a 16-item instrument to measure college students’ general motivation for a healthy lifestyle. Please see S1 File for the details of the CSHM-Q. Results of parallel analysis and exploratory factor analysis showed a three-component structure. The self-focused component has 8 items. It measures autonomous reasons (e.g., pleasure, happiness, etc.) for practicing a healthy lifestyle. The other-focused component has 5 items. It describes externally regulated reasons such as influence or pressure from significant others. The introjected component has 3 items. It indicates the internal struggle during the internationalization process from other-focused health behaviors to self-focused health behaviors. Each item is rated on a five-point Likert-type scale ranging from “strongly disagree” to “strongly agree”. Analyses based on classical test theory have demonstrated adequate psychometric properties. For example, Cronbach’s αs were 0.88, 0.76, and 0.74 for self-focused, other focused, and introjected components, and McDonald’s Omegas were 0.88, 0.76, and 0.75 respectively. In addition, test-retest reliability was good as the intra-class correlation coefficients were 0.88, 0.79, and 0.87 for self-focused, other-focused, and introjected components measured at two timepoints.

### Rasch analysis

Both the RSM and the PCM are appropriate for Rasch analysis with polytomous data. In the RSM, items have the same rating scale structure (i.e., the distances between threshold parameters are maintained across all items), while the PCM allows items’ threshold parameters to wary [15]. In RUMM2030, the PCM is set as the default model. To determine which model should be used, a likelihood ratio test in RUMM2030 can be used to examine the efficiency of the unrestricted parameterization against the restricted rating reparameterization [16, 17]. A significant result supports the use of the PCM, while a nonsignificant result suggests the application of the RSM. In the present study, the PCM was adopted for Rasch analyses based on a significant result of a likelihood ratio test.

Reports of Rasch analyses results usually include: fit indices both at the item level (residual values between ±2.5) and at the scale level (indicated by a non-significant chi-square statistics); local item dependency—residual correlations between any two items >0.2 above the average residual correlations among items [18]; differential item functioning (DIF)—persons on the same ability level respond an item differently just because they are from different demographic groups (e.g., gender or age); unidimensionality—items measure one common underlying construct—supports the legitimate summing of individual item scores into a valid total subscale score [19, 20]), it can be tested by significant t-tests between person estimates calculated separately based on two subsets of items generated by a principal component analysis of the residuals. Less than 5% significant t-tests or the lower bound of the binomial confidence interval overlaps 5% can be considered as a sign of unidimensionality [21]; item category thresholds—thresholds are ordered when individuals’ responses are consistent with their levels of the trait, and good discrimination between two response categories on an item (the thresholds are statistically distinct from each, as indicated by a clear and discernible peak in the category probability curve); estimates of item difficulty and person ability; person separation index (PSI); and scale targeting—a floor or a ceiling effect happens when items could not cover the lowest or highest levels of the latent trait measured in the sample.

A number of strategies—rescoring or collapsing disordered categories, creating ‘super items’ to accommodate locally dependent items, splitting or removing misfit or group-variant items where necessary, etc.—could be used to help reach an approximate agreement between observed scores and model expectations.

In this study, statistical analyses were performed using SPSS version 22 and the Rasch Unidimensional Measurement Model 2030 (RUMM 2030) software [17].

## Results

Because previous parallel analysis and exploratory factor analysis have already revealed a 3-factor structure [5], multidimensionality is present at the scale level, therefore, Rasch analyses were conducted separately for each component. According to the likelihood ratio test, the CSHM-Q items did not meet the requirements of the RSM, thus the PCM was used for subsequent Rasch analyses.

### Self-focused health motivation component

Initial fit to the Rasch model for self-focused component was poor (χ^2^(32) = 57.36, p < 0.01, see Table 2). No mis-fitting items were found as all items’ fit residuals were within the acceptable range (greater than -2.5 or less than 2.5). Testing for local item dependency did not show residual correlations between any two items >0.2 above the average [18]. DIF was not found across age, gender, college year, and family location groups. Multidimensionality was not present as the lower bound of the binomial confidence interval for the pairwise t-tests overlapped 5% (7.14%, CI: 4.6–10.5%, please see Table 2). Disordered thresholds were found for item 3 and item 12 (please refer to S1 Fig in the online supplementary file) and some of their response options were collapsed (please see Table 3). Local item dependency and multidimensionality were examined again and our analyses did not indicate any problem.

**Table 2:** Fit statistics of the CSHM-Q to the Rasch model.

Analysis Name	# of Items	Item Residual		Person Residual		Chi Square		PSI	Cronbach’s Alpha	Unidimensionality
		(Mean ± SD)		Mean ± SD		Value	P			t-test (CI %)
**Self-focused initial**	8	0.08	1.64	-0.56	1.48	57.36	<0.01	0.84	0.91	7.14% (4.6%-10.5%)
**Self-focused final**	8	0.10	1.45	-0.53	1.46	42.68	0.10	0.85	0.91	7.14% (4.6%-10.5%)
**Other-focused Initial**	5	0.96	1.68	-0.48	1.46	48.05	<0.01	0.67	0.68	3.73% (1.9%-6.4%)
**Other-focused final**	5	0.52	1.61	-0.54	1.16	7.83	0.80	0.61	0.60	2.48% (1.1%-5.0%)
**Introjected**	3	0.30	1.65	-0.52	1.02	17.56	0.13	0.78	0.83	3.73% (1.9%-6.4%)

Note: SD = Standard Deviation; p = Probability; PSI = Person Separation Index; CI = Confidence Interval.

**Table 3:** Item fit statistics and scoring strategy for CSHM-Q.

Item description	Location	SE	Fit residual	Chi square	Probability	Scoring strategy
**Self-focused component**						
Q1	0.26	0.09	1.63	7.91	0.09	0-1-2-3-4
Q2	0.05	0.08	-1.45	9.44	0.05	0-1-2-3-4
Q3	0.43	0.09	0.32	2.20	0.70	0-0-1-2-3
Q12	-0.10	0.10	2.26	1.89	0.76	0-0-1-2-3
Q9	-0.50	0.09	-1.26	4.28	0.37	0-1-2-3-4
Q13	-0.15	0.09	-0.72	7.37	0.12	0-1-2-3-4
Q7	0.14	0.08	-0.31	4.15	0.39	0-1-2-3-4
Q11	-0.40	0.09	-1.92	11.18	0.02	0-1-2-3-4
**Other-focused component**						
Q4	-0.23	0.05	2.35	1.23	0.87	0-1-2-3-4
Q8	-0.01	0.04	-0.11	1.61	0.81	0-1-2-3-4
Q16						0-1-2-3-4
Q5	0.24	0.04	-0.69	4.99	0.29	0-1-2-3-4
Q14						0-1-2-3-4
**Introjected component**						
Q6	-0.49	0.08	-1.50	12.73	0.01	0-1-2-3-4
Q15	0.56	0.08	1.76	1.86	0.76	0-1-2-3-4
Q10	-0.07	0.08	0.63	2.97	0.56	0-1-2-3-4

Note: SE = Standard Error; Item 3 and item 12 were rescored; Item 16 and item 8 were added together as a super item, item 5 and item 14 were added together as another super item. Please refer to supplementary file for addition information of the instrument.

**Fig 1:**
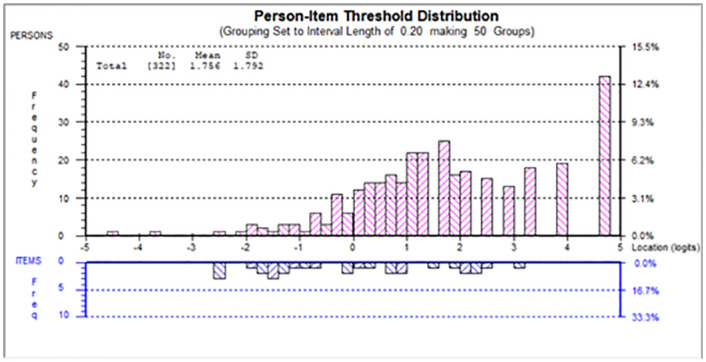
Person-item map for self-focused component.

The modified self-focused component achieved a good model-data fit (χ^2^(32) = 42.68, p = 0.10, see Table 2 self-focused final). All thresholds were ordered correctly. The estimated persons’ ability and items’ difficulty spread reasonably well along the logit continuum in general. Ceiling effect was found, where 46 persons (14.3%) attained the maximum raw total score, floor effect was negligible with only 1 person (0.3%) attained the minimum. Please see Fig 1. Reliability was good, where person separation index = 0.85, and Cronbach’s alpha = 0.91. Remaining items in this component were free from DIFs across different contextual groups.

### Other-focused health motivation component

**Fig 2:**
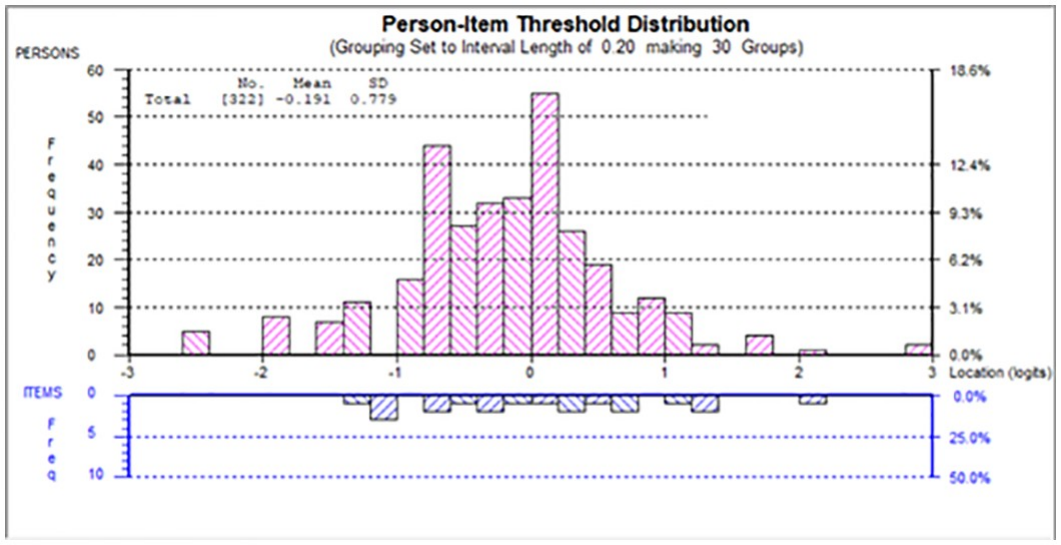
Person-item map for other-focused component.

Initial fit to the Rasch model for other-focused component was poor (χ^2^(20) = 48.05, p < 0.01, see Table 2). Testing for local item dependency (LID) showed that residual correlations between item 8 and item 16 and between item 5 and item 14 are greater than 0.2 above the average [18]. To adjust for the LID, we adopted the ‘super item’ approach by simply adding the LID items together into a larger polytomous item. For example, item 8 (My teachers told me I should have health-promoting lifestyles) and item 16 (I practice health-promoting lifestyles because of the influence from people in public life) were added into a ‘super item’. We tested local item dependency again and did not find any residual correlations between any items >0.2 above the average. The chi-square statistic value (χ^2^(24) = 25.06, p = 0.69, see other-focused final in Table 2) indicated that the items fit the model well. All items’ fit residuals were within the acceptable range. DIF was not found across age, gender, college year, and family location groups. Unidimensionality was supported by significant pairwise t-tests less than 5% (2.48%, CI: 1.1%–5.0%, see Table 2). All thresholds were ordered correctly. The estimated persons’ ability and items’ difficulty spread reasonably well along the logit continuum in general. Mild ceiling effect was found, where 28 persons (8.7%) attained the maximum raw total score, floor effect was negligible with only 6 persons (1.9%) attained the minimum. Please see Fig 2. Person Separation Index = 0.61, and Cronbach’s alpha = 0.60.

### Introjected health motivation component

**Fig 3:**
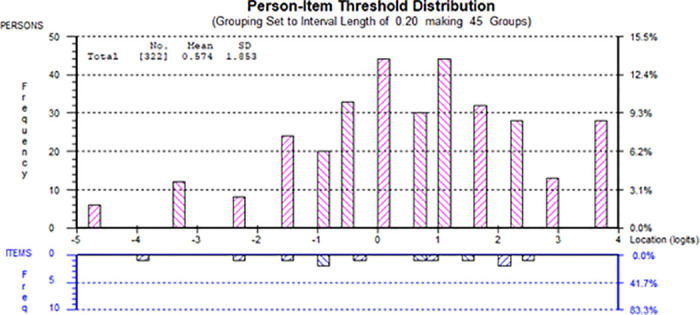
Person-item map for introjected component.

Rasch analyses on items of the introjected component did not reveal any significant problem. For example, the goodness of model fit statistics was acceptable (χ^2^(12) = 17.56, p = 0.13, see Table 2). Fit residuals were all within the acceptable range. Testing for local dependency did not show any positive residual correlations between any items above 0.2. DIF was not found across age, gender, college year, and family location groups. The pairwise t-tests supported unidimensionality (3.73%, CI: 1.9%–6.4%, see Table 2). All thresholds were ordered correctly and were spread reasonably well along the logit continuum. The estimated persons’ ability and items’ difficulty spread reasonably well along the logit continuum. Mild ceiling effect was found, where 28 persons (8.70%) attained the maximum raw total score, and floor effect was negligible with 6 persons (1.86%) attained the minimum. Please see Fig 3. Reliability was good (PSI = 0.78, Cronbach’s alpha = 0.83).

### Scoring strategies

The bifactor model provides a valuable tool for exploring dimensionality related issues [22]. To find out whether all CSHM-Q items can be treated as a single scale (i.e., a total score consisting of all three components), we conducted a bifactor analysis by sub-testing the items from each component, making three subtests in total, and running these as a scale with three items [23]. The RUMM 2030 software would make a bifactor equivalent solution and report the proportion of common variance retained in the data [24]. This proportion—the value of A—should be at least 0.9 if the scale is to be considered unidimensional [25]. In this study, the value of A was 0.73. Therefore, three sub-scores instead of a total sum-score should be used.

We next provided transforming tables to help readers convert the raw score into interval scales (please refer to Tables 4–6). To calculate the raw score, responses of “strongly disagree” were scored 0 and “strongly agree” scored 3. For self-focused component, this scoring strategy yielded total individual scores between 0 and 30. These total raw scores then could be easily converted into interval scales using the location estimates from the Rasch analysis (Table 4). A higher score indicated a higher level of self-focused health motivation.

**Table 4:** The Rasch location estimates and the transformed interval scale for self-focused component.

Raw subscale score	Rasch location	Rasch-converted score
30	4.76	30
29	3.92	27.3
28	3.31	25.3
27	2.87	23.9
26	2.51	22.7
25	2.19	21.7
24	1.91	20.7
23	1.64	19.9
22	1.39	19.1
21	1.15	18.3
20	0.92	17.5
19	0.69	16.8
18	0.47	16.1
17	0.25	15.4
16	0.04	14.7
15	-0.16	14.0
14	-0.36	13.4
13	-0.56	12.7
12	-0.75	12.1
11	-0.94	11.5
10	-1.13	10.9
9	-1.32	10.3
8	-1.51	9.6
7	-1.71	9.0
6	-1.92	8.3
5	-2.15	7.6
4	-2.41	6.7
3	-2.72	5.7
2	-3.11	4.4
1	-3.67	2.6
0	-4.48	0

**Table 5:** The Rasch location estimates and the transformed interval scale for other-focused component.

Raw subscale score	Rasch location	Rasch-converted score
20	2.91	20
19	2.16	17.3
18	1.65	15.4
17	1.32	14.2
16	1.06	13.3
15	0.84	12.5
14	0.65	11.8
13	0.47	11.1
12	0.31	10.6
11	0.15	10.0
10	0.00	9.4
9	-0.15	8.9
8	-0.29	8.4
7	-0.45	7.8
6	-0.61	7.2
5	-0.78	6.6
4	-0.97	5.9
3	-1.21	5.0
2	-1.50	4.0
1	-1.94	2.4
0	-2.60	0

**Table 6:** The Rasch location estimates and the transformed interval scale for introjected component.

Raw subscale score	Rasch location	Rasch-converted score
12	3.78	12
11	2.94	10.8
10	2.26	9.9
9	1.71	9.1
8	1.19	8.4
7	0.67	7.6
6	0.13	6.9
5	-0.42	6.1
4	-0.96	5.3
3	-1.54	4.5
2	-2.22	3.6
1	-3.25	2.1
0	-4.77	0

Using the transformed interval score, we also produced three boxplots for each component separately (please see S2 File). Medians of self-focused and other-focused component between gender groups were at the same level. For other-focused component, a shorter box plot for female participants suggested that female participants had a higher level of agreement with each other compared to males. For introjected component, male participants had a greater median than female participants, suggesting that most male participants had higher levels of introjected health motivation compared to female participants.

## Discussion

Motivation is one of the crucial variables in predicting individual’s health-promoting behaviors [1, 2]. The CSHM-Q is a novel and shorter instrument—a shorter measurement scale is preferable because a longer test usually causes poorer data quality and lower response rate [26]—to measure emerging adults’ motivation for a healthy lifestyle. Test scores of the CSHM-Q showed acceptable validity and reliability based on CTT [5]. This study examined the psychometric properties of the three components of the CSHM-Q separately using a modern psychometric approach—the Rasch analysis.

In this study, unidimensionality at the subscale level was supported by our findings. Summing of individual item raw scores into an interpretable total subscale score is valid because items within each component are all measuring a single latent trait. It should be noted that item 3 (Practicing health-promoting lifestyles is another form of filial piety to my parents) and item 12 (I practice health-promoting lifestyles because I don’t want to get sick) had some disordered thresholds and were rescored. It is advisable to follow a new scoring strategy presented in Table 3. Researchers can further use Tables 4–6 to transform raw scores into interval scales.

The estimated persons’ ability and items’ difficulty spread reasonably well along the logit continuum in general. However, Mild ceiling effect was found across three components of the CSHM-Q. Take the first self-focused component for example, the observed ceiling effect reflected either some of our participants practiced a healthy lifestyle because of self-focused motivations from the psychological perspective, or items for this component were not enough to capture these participants at the highest self-focused levels from the psychometric perspective. More “difficult” items at the highest self-focused levels are needed in the latter case. Based on the consideration that the CSHM-Q was designed for the use in the health education context, and there is not much need to clearly differentiate participants at the highest self-focused or other-focused levels, we tended to believe that the observed ceiling effect would not affect the validity of the instrument.

Differential item functioning (DIF) was examined across gender, age, college year, and family residence groups. To reiterate, DIF occurs when persons on the same ability level respond an item differently just because they are from different groups (e.g., gender or family residence). In other words, a DIF item is the same question biased by different group of people. In this study, all items were DIF-free, which allowed meaningful comparisons across groups. These findings provided a basis for further testing of the DIF using other different samples, and researchers should be cautious when using this instrument for international comparisons.

For the other-focused component, the value of the Person Separation Index (PSI) and the value of Cronbach’s alpha were at the margins of reliability. PSI is calculated using the person estimates in logits whereas Cronbach’s alpha uses the raw scores. When the distribution is normal, PSI is equivalent to Cronbach’s alpha. Although values of PSI and alpha greater than 0.7 are usually considered sufficient [27, 28], some argued that a lower value of alpha is also acceptable [29, 30]. Future effort (e.g., adding more items into the other-focused component) to increase the reliability of this component is needed.

There were several limitations to the present research which also provided directions for future studies. First, this study used a convenience sample from a higher education context in China, which may limit the generalizability of findings to the whole college students population. Second, application of this instrument to other contexts should be performed with caution, and further tests using samples from other cultural groups are needed. In addition, when applying this instrument to other contexts, differential item functioning should be further examined in order to make meaningful comparisons. Finally, other forms of validity, such like convergent and discriminant validity, could be tested in subsequent studies, though it is beyond the scope of the present research.

## Conclusions

In summary, data from each component of the modified CSHM-Q met the expectations of the Rasch model. All 16 items were retained. Each component showed acceptable overall fit, item fit, person fit, and targeting. After modification, no items had disorder thresholds, or showed local dependency or differential item functioning. Total subscale scores can be summed and transformed into interval scales. Evidence from Rasch analysis supported the application of the CSHM-Q as an instrument that can be used to assess emerging adults’ motivation for a healthy lifestyle in health education context.

## Supporting information

S1 Fig Disordered thresholds for item 3 and item 12. (DOCX)

S1 File The instrument used in the study. (DOCX)

S2 File Boxplots for three components. (DOCX)

S3 File (DAT)
