# Supplementary figures and images for: A Rasch analysis of emerging adults’ health motivation questionnaire in higher education context
Source: PLoS One. 2021 Mar 15;16(3):e0248389. doi: 10.1371/journal.pone.0248389 (PMC7959369; doi:10.1371/journal.pone.0248389)

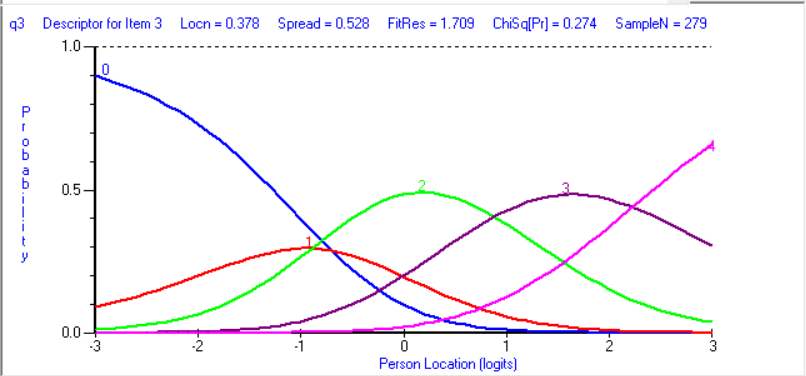


Fig 1 Disordered Thresholds Item 3


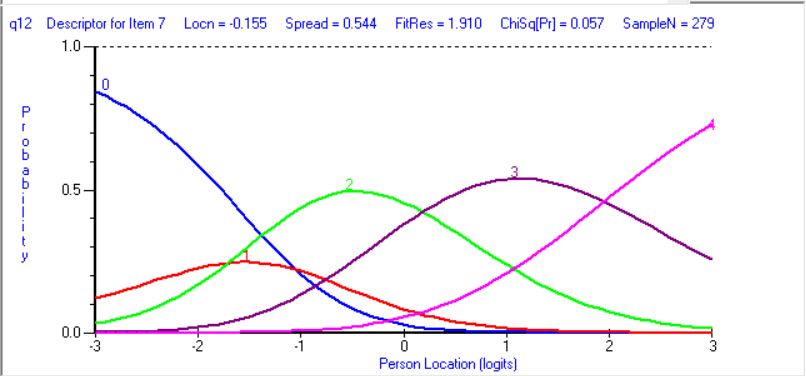


Fig 2 Disordered Thresholds Item 12

Supplement: S1 Fig — (DOCX) [file pone.0248389.s001.docx]

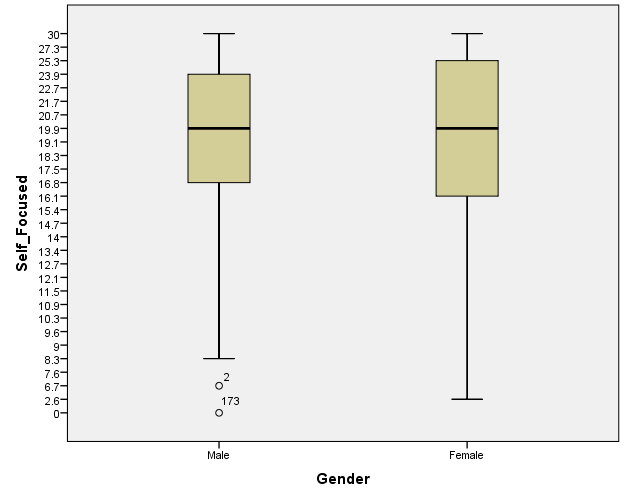


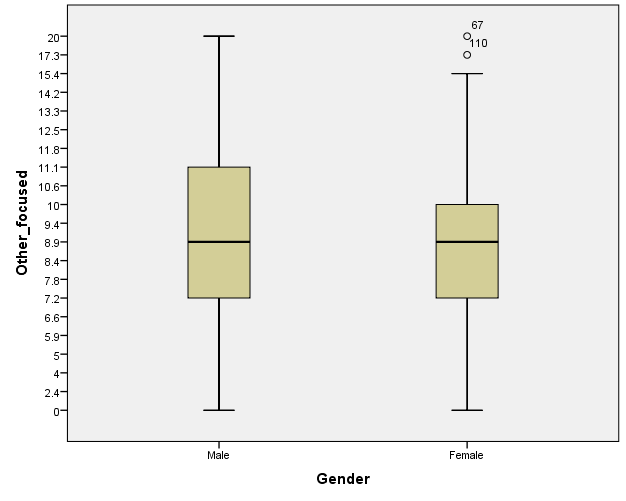


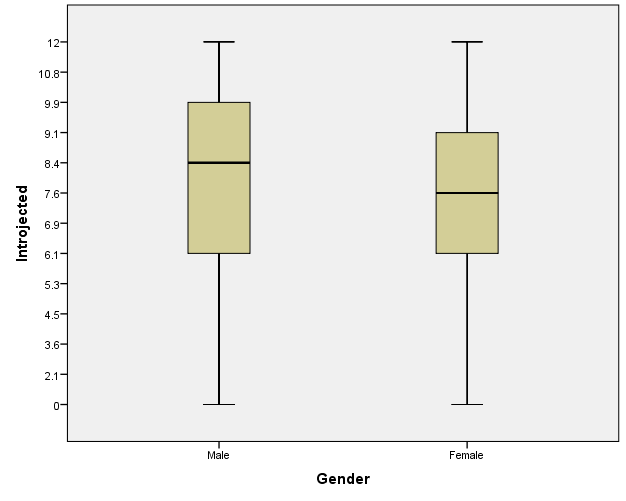


**Fig. Boxplots of three components by gender.**

Supplement: S2 File — (DOCX) [file pone.0248389.s003.docx]
