# Supplementary material for: A Rasch analysis of emerging adults’ health motivation questionnaire in higher education context
Source: PLoS One. 2021 Mar 15;16(3):e0248389. doi: 10.1371/journal.pone.0248389 (PMC7959369; doi:10.1371/journal.pone.0248389)
Supplement: S1 File — (DOCX) [file pone.0248389.s002.docx]

**Health Motivation Questionnaire**

For each question below, please rate the extent to which YOU agree it is your motivation for a healthy lifestyle, from 1 (strongly disagree) to 5 (strongly agree). Please CIRCLE the number that best describes your agreement for each.

|  | **Item Description** | **strongly disagree** | |  | **strongly agree** | |
| --- | --- | --- | --- | --- | --- | --- |
| 1 | I enjoy the process of practicing health-promoting lifestyles | 1 | 2 | 3 | 4 | 5 |
| 2 | I practice health-promoting lifestyles to affect other people positively | 1 | 2 | 3 | 4 | 5 |
| 3 | Practicing health-promoting lifestyles is another form of filial piety to my parents | 1 | 2 | 3 | 4 | 5 |
| 4 | My parents urge me to practice health-promoting lifestyles | 1 | 2 | 3 | 4 | 5 |
| 5 | I practice health-promoting lifestyles because I had health problems in the past | 1 | 2 | 3 | 4 | 5 |
| 6 | I feel regretful when I don't practice health-promoting lifestyles | 1 | 2 | 3 | 4 | 5 |
| 7 | I practice health-promoting lifestyles in order to keep up a good performance on my study | 1 | 2 | 3 | 4 | 5 |
| 8 | My teachers told me I should have health-promoting lifestyles | 1 | 2 | 3 | 4 | 5 |
| 9 | I practice health-promoting lifestyles because I believe health is another form of beauty | 1 | 2 | 3 | 4 | 5 |
| 10 | I feel guilty when I don't practice health-promoting lifestyles | 1 | 2 | 3 | 4 | 5 |
| 11 | I practice health-promoting lifestyles because I believe there are strong connections between health and lifestyle | 1 | 2 | 3 | 4 | 5 |
| 12 | I practice health-promoting lifestyles because I don’t want to get sick | 1 | 2 | 3 | 4 | 5 |
| 13 | I feel pleasure and satisfaction from practicing health-promoting lifestyles | 1 | 2 | 3 | 4 | 5 |
| 14 | I practice health-promoting lifestyles because other people I am familiar with have health problems in the past | 1 | 2 | 3 | 4 | 5 |
| 15 | I despise myself if I fail to practice health-promoting lifestyles | 1 | 2 | 3 | 4 | 5 |
| 16 | I practice health-promoting lifestyles because of the influence from people in public life | 1 | 2 | 3 | 4 | 5 |

健康动机问卷

请仔细阅读下列问题，并勾选最符合自己实际情况的数字。

|  | **Item Description** | **非常不同意** | |  | **非常同意** | |
| --- | --- | --- | --- | --- | --- | --- |
| 1 | 我享受坚持健康的生活方式的过程 | 1 | 2 | 3 | 4 | 5 |
| 2 | 我想通过坚持健康的生活方式对身边的人产生积极影响 | 1 | 2 | 3 | 4 | 5 |
| 3 | 坚持健康的生活方式对我来说是孝顺父母的一种形式 | 1 | 2 | 3 | 4 | 5 |
| 4 | 在父母督促下，我才会坚持健康的生活方式 | 1 | 2 | 3 | 4 | 5 |
| 5 | 自己曾有过健康方面的问题，我才会坚持健康的生活方式 | 1 | 2 | 3 | 4 | 5 |
| 6 | 如果没有坚持健康的生活方式，我会感到后悔 | 1 | 2 | 3 | 4 | 5 |
| 7 | 我希望通过坚持健康的生活方式，提高学习成绩 | 1 | 2 | 3 | 4 | 5 |
| 8 | 在老师建议下，我才会坚持健康的生活方式 | 1 | 2 | 3 | 4 | 5 |
| 9 | 健康的生活方式本身就是一种美 | 1 | 2 | 3 | 4 | 5 |
| 10 | 如果没有坚持健康的生活方式，我会感到内疚 | 1 | 2 | 3 | 4 | 5 |
| 11 | 我想坚持健康的生活方式，是因为我相信生活方式会影响健康 | 1 | 2 | 3 | 4 | 5 |
| 12 | 我想坚持健康的生活方式以避免生病 | 1 | 2 | 3 | 4 | 5 |
| 13 | 坚持健康的生活方式让我得到愉悦和满足 | 1 | 2 | 3 | 4 | 5 |
| 14 | 坚持健康的生活方式，是因为熟识的人出现过健康问题 | 1 | 2 | 3 | 4 | 5 |
| 15 | 如果没有坚持健康的生活方式，我会鄙视自己 | 1 | 2 | 3 | 4 | 5 |
| 16 | 坚持健康的生活方式，是因为受到了公众人物的影响 | 1 | 2 | 3 | 4 | 5 |
